# An Unusual Case of a Mass‐Like Hydroxyapatite Deposition Disease Causing Forearm Flexor Tendon Calcific Tendinitis

**DOI:** 10.1155/crra/8471826

**Published:** 2026-09-28

**Authors:** Aiden Ali, Ronan Kennedy, Robert Henderson, Rajesh Botchu

**Affiliations:** ^1^ University of Birmingham, School of Medicine, Birmingham, UK, birmingham.ac.uk; ^2^ Department of Radiology, Royal Orthopaedic Hospital NHS Foundation Trust, Birmingham, UK, nhs.uk; ^3^ Department of Medical Imaging, University Health Network, Toronto, Canada, uhn.ca

**Keywords:** barbotage, calcific deposition, calcific tendinitis, HADD, hydroxyapatite, ultrasound

## Abstract

Hydroxyapatite deposition disease (HADD) is a relatively common disorder characterised by periarticular deposition of calcium hydroxyapatite, most frequently involving tendons of the glenohumeral joint. Although calcific deposits typically produce a self‐limiting inflammatory response, mass‐like presentations at atypical sites can mimic trauma, infection or soft tissue neoplasia, posing a significant diagnostic challenge. We report an unusual presentation of mass‐like calcific tendinitis within the flexor carpi ulnaris (FCU) tendon of the distal forearm—an atypical location for HADD presenting as a painful soft tissue swelling. Multimodality imaging, including radiography, MRI and CT, was instrumental in characterising the deposit and excluding alternative diagnoses. The patient was treated with ultrasound‐guided barbotage using a large bore needle system, with significant reduction in deposit volume and symptom relief. This case highlights the importance of considering HADD in the differential diagnosis of painful soft tissue masses at atypical sites, where multimodal imaging is essential for accurate characterisation and ultrasound‐guided barbotage represents an effective minimally invasive treatment alternative to surgery.

## 1. Introduction

Soft tissue deposition of calcium hydroxyapatite is a relatively common disorder that typically occurs within periarticular tissues and joint capsules, most commonly precipitating within tendons and tendon sheaths.

Historically there is a myriad of persistent and relapsing pain syndromes caused by calcific deposition in periarticular spaces, which are commonly described as calcific tendinitis and periarthritis; however, following isolation of crystalline calcium hydroxyapatite responsible, the descriptive pathological term hydroxyapatite deposition disease (HADD) has been used [1, 2].

Although typically self‐limiting, the presence of HADD at specific locations can cause persistent pain and reactive inflammatory response to masquerade as infection, trauma and neoplasia [3, 4].

## 2. Case Report

We present the case of a 42‐year‐old right hand dominant female with a slow progressive painful swelling in her distal forearm. No preceding history of trauma. On examination, there was a firm and mildly tender lump at the ulnar aspect of her distal forearm not tethered to the overlying skin. The lump would move with both active and resisted wrist flexion/extension.

The forearm radiograph (Figure 1) identified a globular mass‐like calcification situated in the outer volar aspect of the distal forearm, level with the distal ulnar shaft to the ulnar styloid tip. The well‐circumscribed calcification tapers at both proximal and distal margins. Minor swelling is noted within the volar forearm soft tissues proximal to the lesion, with localised indistinction at the muscle‐subcutaneous fat interface. The underlying bone is normal.

**Figure 1:**
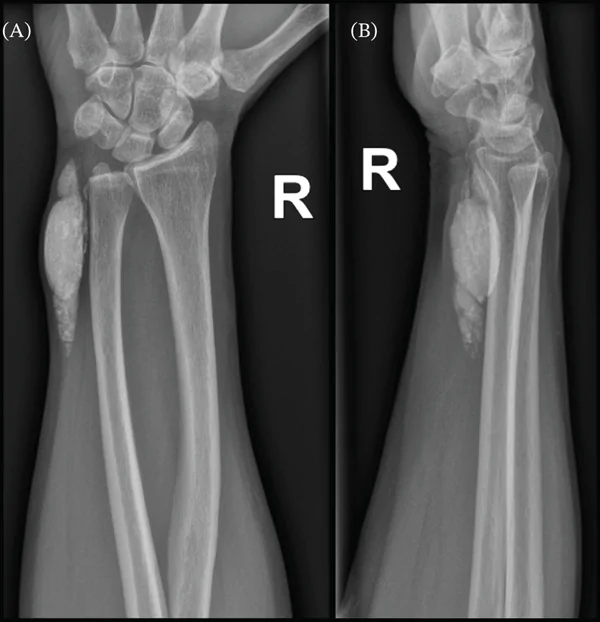
(A,B) Well‐circumscribed globular and moss‐like deposition of soft tissue calcification at the volar‐ulnar aspect of the distal forearm, level with the distal ulna. Normal appearance of the underlying cortex.

On further characterisation with MRI, the globular calcification correlates to low signal on both T1‐weighted and short tau inversion recovery (STIR) sequences (Figures 2A–G). Multiplanar sequences confirm the mass is contiguous with the distal flexor carpi ulnaris (FCU) tendon a short distance proximal to its distal attachment at pisiform. Axial images (Figure 2E,F) show a uniformly low signal mass centred within an expanded distal FCU tendon toward its myotendinous junction. The distal ulnar neurovascular bundle is contacted but no significant compression or distortion. The surrounding soft tissues and underlying ulna are normal.

**Figure 2:**
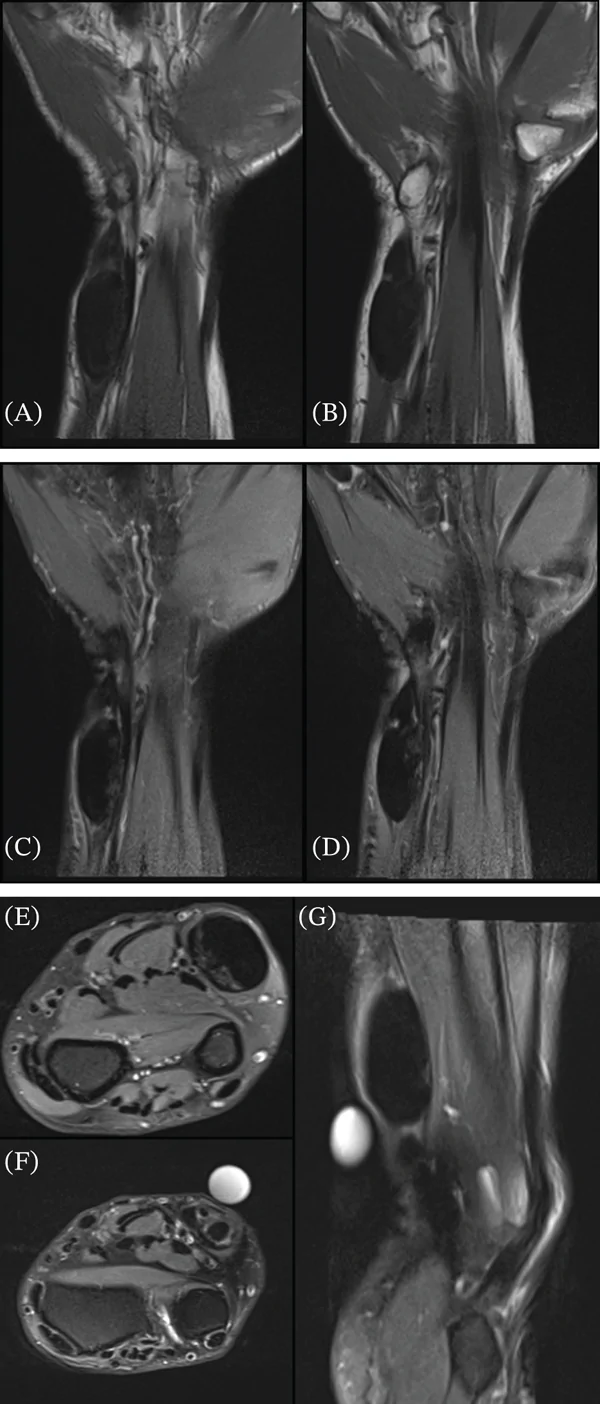
Sequential (A,B) coronal T1‐weighted and (C–G) multiplanar short tau inversion recovery (STIR) demonstrate an elongated globular low signal structure associated with an expanded distal flexor carpi ulnaris (FCU) tendon. Low signal material tapers toward its distal insertion to pisiform with localised fragmentation at both proximal and distal margins. Faint and indistinct high signal halo although no significant surrounding soft tissue oedema.

Targeted CT (Figure 3B–D) further characterises the confluent globular calcification mass with an incomplete eggshell margin and intrinsic speckled densities; with mean density 1038 Hounsfield units (HU) and range 775–1639 HU (Figure 3C). This correlates to the diagnostic radiograph (Figure 3A) and axial STIR MRI sequence discussed above (Figure 3E).

**Figure 3:**
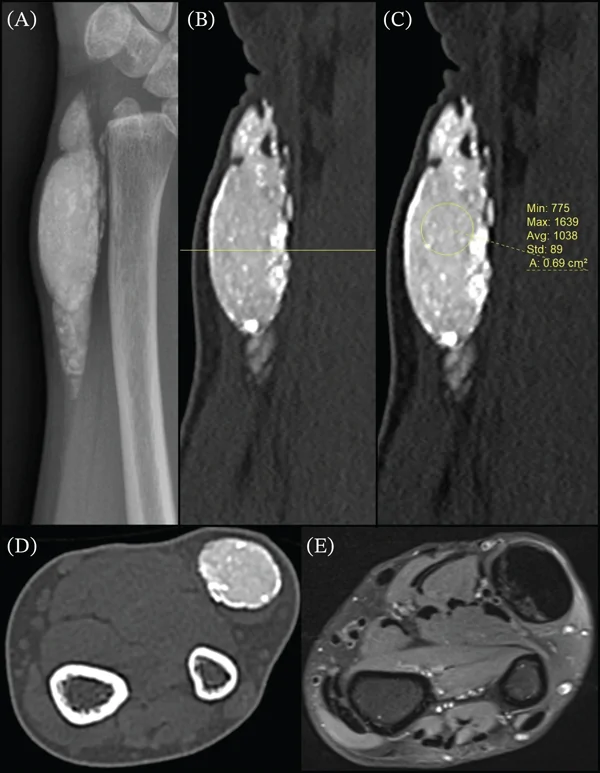
Correlating (A) the initial radiograph, the confluent calcific mass has an incomplete eggshell margin with intrinsic speckled hyperdensities on (B–D) sagittal and axial reformatting. Mean density 1038 Hounsfield units (HU), with range 775–1639 HU is consistent with calcification; this further correlates with the (E) globular low signal on axial STIR sequence.

Proceeding onto image‐guided biopsy, preprocedure ultrasound demonstrates a hyperechoic mass with poor characterisation of the internal characteristics due to highly reflective surface and posterior acoustic shadowing (Figure 4A, B, E and F). On Doppler signal application, there is a comet tail artefact consistent for calcinosis/calcification [3] (Figure 4C,D). Sampling was then performed under continuous ultrasound guidance (Figure 5A), yielding friable grainy fragments of a creamy white substance.

**Figure 4:**
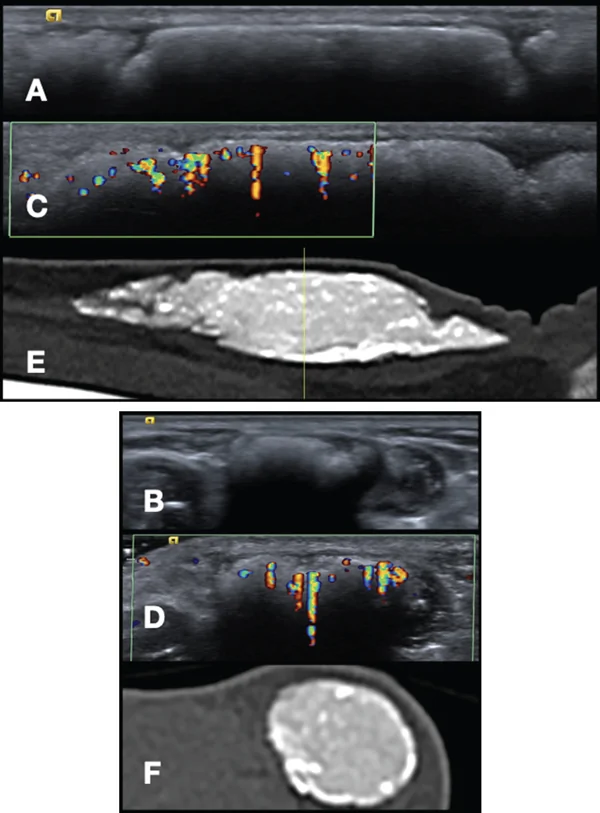
(A,B) Prebiopsy/treatment ultrasound demonstrated a confluent echogenic globular structure with macrolobulated eggshell margin and posterior acoustic shadowing. Following (C, D) Doppler application there was comet tail artefact consistent with calcification, and correlative to the (E,F) CT findings.

**Figure 5:**
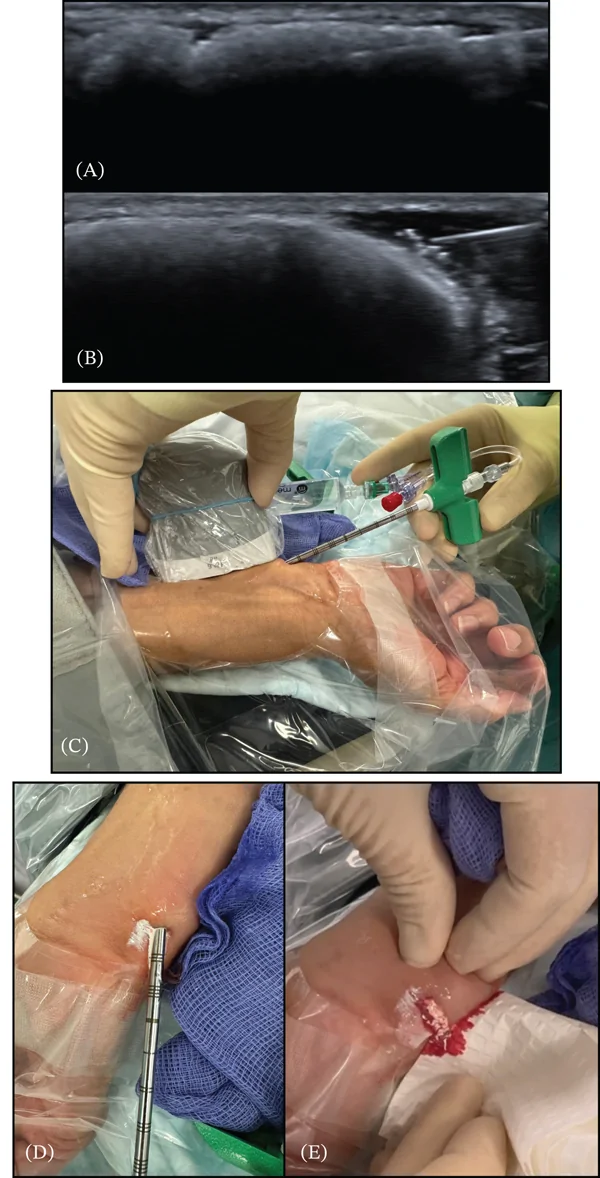
Ultrasound images demonstrating (A) biopsy sampling and (B) barbotage needle placement; photo of procedural technique with ultrasound transducer placement and alignment to the (C) large bore needle system; photos demonstrating the (D) discharge of milky white material bypassing the needle and then self‐discharging and with (E) massage following needle removal.

There was a high preprocedure index of suspicion for calcific deposition, and with full informed consent obtained from the patient, we immediately proceeded to postbiopsy ultrasound‐guided barbotage. Because of the lesion size and large volume of expected calcific debris, a large bore 8‐gauge T‐Lok bone biopsy system was employed (Figure 5B,C following generous infiltration of ropivacaine 0.75% local anaesthetic. Further discharge of the creamy white grainy material was observed bypassing the large bore biopsy needle (Figure 5D), and then self‐discharging following needle removal on procedure completion (Figure 5E). Despite the relative success of the barbotage, given the extent of the calcific tendinitis, she will likely require a repeat procedure.

On microscopic pathological evaluation, there were no cellular components or evidence of giant cell reaction. No other common crystallopathy such as gout, oxalate or pyrophosphate deposition was identified.

## 3. Discussion

This report describes an unusual presentation of HADD within the lower forearm involving the distal tendon segment of FCU, a muscle located superficially in the forearm flexor compartment. Arising primarily from the medial epicondyle with its aponeurotic sheath from the medial olecranon to the mid ulnar shaft, the muscle condenses to the tendon within the lower third of forearm before distal tendinous insertion to pisiform with slips to the hook of hamate and 5th metacarpal base.

Historically, there is a myriad of persistent and relapsing pain syndromes caused by calcific deposition in periarticular spaces, which are commonly described as calcific tendinitis and periarthritis; however, following isolation of crystalline calcium hydroxyapatite responsible, the descriptive pathological term HADD [1, 2] has been used. On the contrary, calcific deposition arthropathy is conventionally associated with the pathological process of calcium pyrophosphate deposition disease (CPPD) to synovium, hyaline cartilage and periarticular tissues.

The pathophysiology of HADD follows a recognised sequence of stages [5, 6]. In the precalcific phase, tenocytes undergo fibrocartilaginous metaplasia, predisposing the tendon to calcium crystal deposition. The calcific stage comprises three subphases: The formative phase, during which calcium hydroxyapatite crystals precipitate to form chalk‐like deposits with relatively well‐defined radiological margins; the resting phase, in which deposits consolidate and may remain quiescent with absent or minimal symptoms; and the resorptive phase, during which vascular ingrowth facilitates phagocytosis by macrophages and polymorphonuclear cells, disrupting crystals into surrounding soft tissues and triggering an acute inflammatory reaction [3, 5]. The calcific‐resorptive phase is followed by the postcalcific phase, where the calcific tissue is replaced by granulation tissue [6]. The imaging appearances in this case (including incomplete eggshell margin, speckled internal densities on CT and the semiliquid consistency of material recovered at barbotage) are characteristic of the resorptive phase described above. This acute, painful, inflammatory reaction can mimic other diseases and therefore lead to initial misdiagnoses such as septic arthritis and gout, both of which can be easily differentiated from HADD through characteristic presence of calcific deposits shown on radiographs [7]. Furthermore, involvement of surrounding tendons such as the FCU detailed in this case may mimic symptoms of tendinitis from overuse injuries and lead to more conservative initial management without prompting further investigations and imaging until a later stage [8].

The most common location for HADD is the glenohumeral joint [9], which supports early theories for preferential involvement of joints with larger range of motion [10] and watershed areas with relative hypovascularity such as the supraspinatus tendon [11]. Despite these suppositions however, recent reports of HADD occurring in various unusual locations such as the carpal tunnel, superior extensor retinaculum of the ankle, lateral collateral ligament of the knee, longus colli, pronator quadratus tendon and lateral patellofemoral ligament expansion of the quadriceps tendon insertion to the patella [6, 12–16] lend to an alternative pathogenesis. This is further supported by this case report, which highlights another unusual location for HADD to develop.

Despite the apparently rare occurrence of HADD outside of the glenohumeral joint, this case report highlights the importance of including HADD as a differential when assessing nonspecific wrist pain and inflammation [6]. Inclusion of HADD as a differential alongside conditions such as gout, septic arthritis and tendinitis may prevent misdiagnosis and initial mismanagement of HADD, which can easily be distinguished from these former conditions through radiography and follows a different treatment pathway, as shown in this report. Beyond diagnosis, this case also illustrates the use of ultrasound‐guided barbotage as a safe, minimally invasive treatment for symptomatic calcific tendinitis. The technique involves sonographic localisation of the deposit, percutaneous needle puncture and lavage with saline to mechanically fragment and aspirate the hydroxyapatite material, reducing volume of deposits and stimulating the natural resorptive process. Performed under local anaesthetic as a day‐case procedure, barbotage provides an alternative to surgery whilst preserving the option for surgical intervention in refractory cases. Serafini et al. demonstrated sustained symptomatic and radiological improvement in both the short and long term (10‐year follow‐up) following ultrasound‐guided barbotage, supporting its role as a robust first‐line interventional option [17]. In the present case, the unusually large deposit necessitated the use of a large bore biopsy system; although initial barbotage achieved significant reduction in deposit volume and symptomatic improvement, repeat treatment may be required to achieve complete resolution.

## Funding

No funding was received for this manuscript.

## Conflicts of Interest

The authors declare no conflicts of interest.

## Data Availability

The data that support the findings of this study are available on request from the corresponding author. The data are not publicly available due to privacy or ethical restrictions.
